# Changes in Dairy Food and Nutrient Intakes in Australian Adolescents 

**DOI:** 10.3390/nu4121794

**Published:** 2012-11-22

**Authors:** Carole E. Parker, Wendy J. Vivian, Wendy H. Oddy, Lawrence J. Beilin, Trevor A. Mori, Therese A. O’Sullivan

**Affiliations:** 1 School of Exercise and Health Science, Edith Cowan University, Joondalup, Western Australia, 6027, Australia; Email: carolep@our.ecu.edu.au (C.E.P.); wvivian@our.ecu.edu.au (W.J.V.); 2 Telethon Institute for Child Health Research, Centre for Child Health Research, University of Western Australia, West Perth, Western Australia, 6005, Australia; Email: wendyo@ichr.uwa.edu.au; 3 School of Medicine and Pharmacology Royal Perth Hospital Unit, The University of Western Australia, Perth, 6000, Australia; Email: lawrie.beilin@uwa.edu.au (L.J.B.); trevor.mori@uwa.edu.au (T.A.M.)

**Keywords:** dairy, dietary intake, adolescent, teenage, Raine study, calcium, milk, yoghurt, cheese

## Abstract

Dairy nutrients, such as calcium, are particularly important in adolescence, a critical time for growth and development. There are limited Australian data following individuals through adolescence, evaluating changes in dairy nutrient and dairy product consumption. We used a validated food frequency questionnaire to investigate consumption in adolescents participating in both the 14 and 17 year follow-ups of the Western Australian Pregnancy Cohort (Raine) Study. Most adolescents did not reach age and gender specific recommended daily intakes for calcium or magnesium at 14 years, and this decreased as they aged to 17 years (from 33.0% to 29.2% meeting for calcium, *P* < 0.05, and from 33.6% to 20.5% meeting for magnesium, *P* < 0.01). Mean intakes of calcium, potassium, riboflavin and vitamin A also decreased with age (*P* < 0.01). Mean dairy intake decreased from 536 ± 343 g/day to 464 ± 339 g/day (*P* < 0.01), due mostly to a decrease in regular milk, although flavoured milk consumption increased in boys. Cheese and butter were the only products to show a significantly increased consumption over the period. Girls decreased from 2.2 to 1.9 serves/day of dairy, while boys remained relatively steady at 2.9 to 2.8 serves/day. Our findings suggest that dairy product consumption decreases over adolescence. This may have implications for bone mass, development and later health.

## 1. Introduction

Dairy products such as milk, cheese and yoghurt are an important source of essential micronutrients including calcium, riboflavin, phosphorus, potassium, magnesium, zinc, vitamin A and vitamin B_12_ [1]. They also provide a combination of protein, carbohydrate, and fat. Nutrients from dairy products are well known for their role in building and maintaining strong bones [2], although meta-analyses of different populations show varied results in the area of dairy and bone health. Increased dietary calcium and dairy products have been shown to significantly improve total bone mineral content in children with initially low intakes [3], and positive benefits of calcium on bone mineralization in children and adolescents have been found in nine out of ten randomized controlled trials in a meta-analysis [4]. Despite this, the latter analysis found no consistent benefit when all types of studies were considered [4], and an analysis examining milk and hip fracture risk in adults was also inconclusive [5]. However dairy nutrients are also reported to be important for immune and nervous systems, red blood cell production, eyesight, muscle and nerve function, skin maintenance and wound healing [6]. In terms of chronic disease, higher dairy intakes are also associated with a reduction in risk for all cause deaths, cardiovascular disease and diabetes [7]. Hence, dairy products are recommended as part of a healthy diet in childhood, adolescence [8] and adulthood [9].

Adequate intake of nutrients found in dairy products, such as calcium, is particularly important in adolescence as this is a critical time for growth and accumulation of bone mass [10,11], with 40% of total bone mass development occurring during this period [12]. Bone turnover is high and bone accrual lags due to increases in height and weight, with peak bone mass usually attained by 20 years of age [13,14]. This increase in nutrient requirements for growth coincides with increasing autonomy of the adolescent to make their own food and drink choices [15], and the establishment of eating patterns that track into adulthood [16]. Increasing independence, peer acceptance, more time spent at school or other activities, and self image contribute to food choices made at this time, setting the scene for long term health outcomes [17,18]. 

Despite the importance of dairy nutrients, the 2007 Australian National Children’s Nutrition and Physical Activity Survey indicated the majority of Australian adolescents do not consume the recommended intakes of some of these nutrients, in particular calcium [19]. Dieting is one factor that has been strongly associated with low consumption of dairy foods [20]. Girls appear to be at a higher risk than boys of not meeting their dietary calcium requirements, and consumption of dairy foods is often reported as low in adolescent groups [19,21,22,23]. Analysis of dairy products from the Australian National Children’s Nutrition and Physical Activity Survey indicated that milk products and dishes contributed between 32% and 60% of dietary calcium across childhood and adolescence, highlighting their importance in the diet [24]. To our knowledge, there is a lack of Australian data following the same individuals through adolescence evaluating how nutrient intakes and dairy product consumption change over time. 

This study tracks the dietary intakes of dairy products and associated nutrients of adolescents participating in the Western Australian Pregnancy Cohort (Raine) Study. The same subjects were followed from 14 to 17 years of age. We hypothesize that intake of dairy products and associated nutrients decreases from 14 to 17 years, and that greater decreases are associated with female gender, lower socio-economic status and higher weight category. 

## 2. Subjects and Methods

### 2.1. Subjects

The Raine Study is a longitudinal observational study that commenced in 1989 when 2900 mothers were recruited from King Edward Memorial Hospital and local clinics in Perth, Australia. Details of recruitment have been previously published, with 2868 babies available for follow-up [25]. Children were assessed at birth, 1, 2, 3, 5, 8, 10, 14 and 17 years of age. This study reports on data collected at the 14 and 17 year follow-ups, with collection at each follow-up occurring over a three year period commencing in 2003 and 2006, respectively. From the original cohort of 2868 live births, 1631 adolescents completed the FFQ in the 14 year follow-up and 1009 completed the FFQ in the 17 year follow-up. A total of 860 adolescents (46% male, 89% Caucasian) who completed FFQs at both the 14 and 17 year follow-ups were included in this study, after 26 were excluded for implausible energy intakes. The ethics committees of King Edward Memorial Hospital for Women and Princess Margaret Hospital for Children granted ethics approval for the study. Informed consent for participation was obtained from the participant’s parent or guardian, and the participant.

### 2.2. Dietary Data Collection

At both the 14 and 17 year follow-ups, dietary intakes were assessed using a self reported food frequency questionnaire (FFQ) booklet developed by the Commonwealth Scientific and Industrial Research Organization (CSIRO) Adelaide, Australia [26]. This semi-quantitative FFQ asked about frequency of consumption in relation to standard serve sizes and collected information on 212 foods, mixed dishes, and beverages, including items popular amongst adolescents [27]. Caregivers assisted with completion of the booklets. The FFQs were checked by a research nurse and discrepancies clarified with the adolescent/caregiver [27].

### 2.3. Weight Status and Socio-Economic Status

Height was measured to the nearest 0.1 cm using a Holtain Stadiometer, and weight to the nearest 100 g using a Wedderburn Digital Chair Scale. Body mass index (BMI) was calculated as weight (kg)/(height (m))^2^. Age and gender specific BMI cut offs were used to determine underweight, normal weight, overweight and obesity [28,29]. The Socio-Economic Indexes for Areas (SEIFA) based on 2006 Australian Bureau of Statistics census data for Index of Education and Occupation, ranked residential postcodes into decile ratings as a measure of socio-economic status, with lower deciles being relatively disadvantaged compared to higher deciles [30]. 

### 2.4. Data Analysis

Using the FFQ data, dairy consumption was calculated for core dairy foods which included total milk, yoghurt, cheese, custard, and non-core dairy foods butter, cream and ice cream. Total milk comprised of flavored milk, milkshakes and smoothies in addition to plain milk. Contributions to milk, butter and cheese from mixed dishes such as milk puddings, mornay dishes and pizza were included after analysis with FoodWorks^®^ Professional 2009 dietary software (Xyris Software Pty Ltd., Queensland, Australia). Recipes were standardised against “Cookery the Australian Way” [31] and the “Taste Australia” website [32], with dairy content determined and added to the applicable category. Dairy foods were categorised into either regular or reduced fat dairy, which were either specified as such in the FFQ or calculated based on FoodWorks analysis or nutrient information panels. 

Serve sizes were based on the Australian Guide to Healthy Eating for milk (250 mL), cheese (40 g), yoghurt (200 g) and custard (250 mL) [33]. A serve size was estimated to contain 300 mg calcium (range 252–350 mg), and this was used to calculate equivalent serve sizes for non-core dairy foods based on calcium content. The recommended daily intake for serves of dairy that we used to compare our data was three serves per day, based on the Australian Guide to Healthy Eating “Healthy Diet example A” for children aged 12–18 years [33].

Data were analysed with Predictive Analytics Software (PASW) for Windows, version 18.0, 2009 (SPSS Inc., IBM, Chicago, IL, USA). Descriptive statistics including mean and standard deviation were used to report data within each age group and gender. Data analyses were conducted on each age cohort for intakes of nutrients including calcium, magnesium, phosphorus, potassium, zinc, vitamin B12, vitamin A, riboflavin, protein, saturated, monounsaturated and polyunsaturated fats and were compared to Australian Nutrient Reference Values for Recommended Daily Intakes (RDI) or Adequate Intakes (AI) if RDI were not available [34]. The Estimated Average Requirement (EAR) was also calculated for each nutrient. EAR describes the daily nutrient level required to meet the needs of half the healthy individuals of a gender and life stage [34]. 

Subjects were excluded due to implausible intake energy data, defined as less than 3000 kJ or greater than 20,000 kJ [35]. Paired sample *t*-tests were used to assess changes in individual intakes over time, which was appropriate for both normal and non-normal distributions given our large sample size [36]. Crosstab analyses identified percentages of adolescents meeting the RDI for each nutrient and were also used to categorise the spread of dairy nutrients consumption across genders and age groups. The McNemar test was performed to identify whether the proportion of adolescents meeting recommended intakes were different between 14 and 17 year follow-ups. Analysis of variance was used to assess associations between BMI, socio-economic status (SES) and change in dairy intake. *P* values less than 0.05 were considered statistically significant.

## 3. Results

### 3.1. Subjects

The mean age of participants included in this study at the 14 year follow-up was 14.0 ± 0.2 years with a range of 13.0–14.9 years, while the mean age at the 17 year follow-up was 16.9 ± 0.2 years with a range of 15.8–18.3 years. The majority of participants were of normal weight at the 14 year follow-up (69.4%), followed by overweight (18.0%), underweight (6.9%) and obese (5.7%). The majority of participants were in the highest three deciles of SEIFA (52.3%), followed by the middle four deciles (33.7%) and the lowest three deciles (14.0%).

### 3.2. Intake of Dairy Nutrients

Micro and macronutrient intakes of interest for the 14 and 17 year follow-ups are displayed in Table 1. At 14 years, the majority of both boys and girls did not reach the RDI for calcium or magnesium, and the proportion meeting the RDI went on to decrease at 17 years for both these nutrients, significantly for magnesium (both genders) and for calcium (girls only). Although total energy intake was not significantly different for the group from 14 to 17 years, mean intakes of potassium, riboflavin, vitamin A and fats decreased. Mean intakes of vitamin B_12_, zinc, energy, and protein significantly increased for boys, while no nutrients significantly increased for girls. The majority of this cohort met the EAR for most of the nutrients, with exceptions of calcium and magnesium. For calcium, 41.4% of girls at 14 years and 30.6% at 17 years met the EAR, while 62.7% of boys at 14 years and 59.9% at 17 years met the EAR. Similarly, for magnesium, 50.1% of girls at 14 years and 34.1% at 17 years met the EAR, while 57.9% of boys at 14 years and 46.6% at 17 years met the EAR.

### 3.3. Intake of Dairy Products by Grams

Intake of dairy products by type at 14 and 17 years is shown in Table 2, for the total group and also separately for those considered to be “consumers” (adolescents who reported consuming an average of at least one gram per day of the product). Total dairy intake significantly decreased for both boys and girls, due mostly to a decrease in consumption of regular milk, although flavoured milk consumption increased in boys. Cheese and butter were the only dairy products to show a significantly increased consumption over the adolescent period. Milk was the product consumed by the highest proportion of adolescents, followed by cheese and ice cream.

**Table 1:** Micro and macronutrient intakes for 14 and 17 year Raine study follow-up participants compared to Recommended Daily Intake (RDI) according to gender and age in years (y).

		14 year follow-up (aged 13–15 y)								17 year follow-up (aged 16–18 y)							
Nutrients	One dairy serve ^a^	Total (*n* = 860)		Girls (*n* = 461)			Boys (*n* = 399)			Total (*n* = 860)		Girls (*n* = 461)			Boys (*n* = 399)		
		Mean ± SD	% meet RDI	RDI ^b^	Mean ± SD	% meet RDI	RDI	Mean ± SD	% meet RDI	Mean ± SD	% meet RDI	RDI	Mean ± SD	% meet RDI	RDI	Mean ± SD	% meet RDI
**Calcium (mg)**	300	1154 ± 525	33.0	1300	1030 ± 480	23.4	1300	1298 ± 539	44.1	1088 ± 592 **	29.2 *	1300	913 ± 465 **	17.6 *	1300	1291 ± 655	42.6
**Potassium (mg)**	318	3674 ± 1217	70.2	AI ^c^*13 y: *2500 *14–15 y: *2600	3476 ± 1217	76.4	AI ^c^*13 y: *3000 *14–15 y: *3600	3903 ± 1177	63.2	3408 ± 1276 **	56.9 **	2600	3095 ± 1113 **	63.3 **	3600	3771 ± 1355	49.4 **
**Phosphorus (mg)**	251	1599 ± 561	70.6	1250	1456 ± 525	59.2	1250	1765 ± 558	83.7	1564 ± 657	63.8 **	1250	1343 ± 526 **	51.2 **	1250	1819 ± 701	78.4 *
**Magnesium (mg)**	23.6	309 ± 102	33.6	*13 y: *240 *14–15 y: *360	288 ± 98.9	31.7	*13 y: *240 *14–15 y: *410	335 ± 100	35.8	303 ± 115	20.5 **	360	269 ± 96.8 **	14.8**	410	342 ± 122	27.1 **
**Riboflavin (mg)**	0.47	2.37 ± 0.95	93.3	*13 y: *0.9 *14–15 y: *1.1	2.13 ± 0.85	91.8	*13 y: *0.9 *14–15 y: *1.3	2.65 ± 0.98	95.0	2.24 ± 1.03 **	88.0 **	1.1	1.91 ± 0.84 **	84.9 **	1.3	2.63 ± 1.10	91.6
**Vitamin A ^d^ (μg)**	86.7	1219 ± 582	83.7	*13 y: *600 *14–15 y: *700	1176 ± 610	87.0	*13 y: *600 *14–15 y: *900	1269 ± 543	79.9	1091 ± 802 **	67.5 **	700	1030 ± 716 **	70.6 **	900	1161 ± 887 *	63.7 **
**Vitamin B12 (mg)**	n/a	4.34 ± 2.12	88.7	*13 y: *1.8 *14–15 y: *2.4	3.93 ± 2.10	84.2	*13 y: *1.8 *14–15 y: *2.4	4.81 ± 2.03	94.0	4.41 ± 2.76	83.2 **	2.4	3.70 ± 2.34 **	75.1 **	2.4	5.23 ± 2.98 *	92.7
**Zinc (mg)**	1.12	12.5 ± 4.1	78.5	*13 y: *6 *14–15 y: *7	11.5 ± 3.8	90.9	*13 y: *6 *14–15 y: *13	13.5 ± 4.1	64.2	12.2 ± 4.8	68.7 **	7	10.5 ± 4.0 **	81.6 **	13	14.1 ± 4.9 *	53.4 **
**Energy (MJ)**	842	9.42 ± 2.89	n/a	n/a	8.74 ± 2.78	n/a	n/a	10.20 ± 2.82	n/a	9.35 ± 3.37	n/a	n/a	8.00 ± 2.64 **	n/a	n/a	10.90 ± 3.47 **	n/a
**Protein (g)**	9.67	92.7 ± 28.9	95.1	*13 y: *35 *14–15 y: *45	85.3 ± 27.3	96.7	*13 y: *40 *14–15 y: *65	101 ± 28.4	93.2	92.2 ± 36.1	89.5 **	45	79.1 ± 28.8 **	90.9 **	65	107.3 ± 37.9 **	88.0 **
**Saturated fat (g)**	5.77	39.0 ± 15.8	n/a	n/a	35.7 ± 14.5	n/a	n/a	42.8 ± 16.5	n/a	37.1 ± 18.3 **	n/a	n/a	30.7 ± 14.4 **	n/a	n/a	44.6 ± 19.6	n/a
**Mono-unsaturated fat (g)**	2.13	30.8 ± 11.2	n/a	n/a	28.5 ± 10.3	n/a	n/a	33.4 ± 11.7	n/a	28.7 ± 12.4 **	n/a	n/a	24.1 ± 9.73 **	n/a	n/a	34.0 ± 13.2	n/a
**Poly-unsaturated fat (g)**	0.27	13.5 ± 6.5	n/a	n/a	12.8 ± 6.1	n/a	n/a	14.2 ± 6.9	n/a	11.7 ± 6.5 **	n/a	n/a	10.4 ± 5.7 **	n/a	n/a	13.3 ± 7.1 *	n/a

* *P* < 0.05 significant difference from 14 y, ** *P* < 0.01 significant difference from 14 y; ^a^ Nutrients provided by standard serve of dairy, based on average values of cheese (40 g), yogurt (200 g), regular milk 3.5% fat (250 mL) and custard (250 mL); ^b^ RDI = Recommended Daily Intake (RDI) according to gender and age in years [34]; ^c^ AI = Adequate Intake—the average daily nutrient intake, assumed to be adequate for a group of healthy people [34]; ^d^ Vitamin A in retinol equivalents.

**Table 2:** Daily intake of dairy products for 14 and 17 year Raine study follow-up participants for the total group and those who are consumers (>1 g/day).

Dairy product	14 year follow-up			17 year follow-up		
	Total (*n* = 860) Mean ± SD	Boys (*n* = 399) Mean ± SD	Girls (*n* = 461) Mean ± SD	Total Mean ± SD	Boys (*n* = 399) Mean ± SD	Girls (*n* = 461) Mean ± SD
**Total dairy (g)**	523 ± 326	622 ± 349	437 ± 279	452 ± 330 **	570 ± 372 **	350 ± 246 **
**Consumers (%)**	99.9	100	99.8	99.8	99.7	99.8
**Consumer intake (g) ^#^**	523 ± 326	622 ± 349	438 ± 278	453 ± 329	571 ± 371	350 ± 246
**Total milk (g)**	424 ± 307	517 ± 335	343 ± 254	368 ± 309 **	479 ± 353 *	272 ± 225 **
**Consumers (%)**	98.7	99.2	98.3	97.3 *	98.2	96.5
**Consumer intake (g) ^#^**	429 ± 305	521 ± 333	349 ± 252	378 ± 307	488 ± 350	281 ± 223
**Regular milk (g)**	205 ± 297	269 ± 362	149 ± 212	153 ± 260 **	223 ± 18 **	92 ± 176 **
**Consumers (%)**	55.0	55.1	54.9	48.7 **	53.4	44.7 **
**Consumer intake (g) ^#^**	373 ± 313	488 ± 361	273 ± 221	314 ± 298	419 ± 329	206 ± 214
**Reduced fat milk (g)**	150 ± 253	167 ± 267	136 ± 238	138 ± 240	160 ± 285	118 ± 191
**Consumers (%)**	42.2	40.9	43.4	42.8	39.8	45.3
**Consumer intake (g) ^#^**	357 ± 279	410 ± 276	314 ± 274	323 ± 274	403 ± 326	262 ± 207
**Skim milk (g)**	17.6 ± 85.9	20.8 ± 98.8	14.8 ± 72.9	23.8 ± 101	19.9 ± 97.8	27.2 ± 104 *
**Consumers (%)**	6.3	6.0	6.5	9.5 **	7.3	11.5 **
**Consumer intake (g) ^#^**	280 ± 211	346 ± 226	227±185	250 ± 228	275 ± 252	237 ± 215
**Milkshakes (g)**	22.1 ± 39.2	24.7 ± 41.7	19.8 ± 36.8	17.1 ± 37.1 **	23.3 ± 47.1	11.7 ± 24.3 **
**Consumers (%)**	56.3	57.8	54.8	48.2 **	51.7	45.0 **
**Consumer intake (g) ^#^**	39.3 ± 45.4	42.3 ± 47.3	36.6 ± 43.5	36.2 ± 47.1	45.7 ± 57.6	26.5 ± 30.7
**Flavored milk (g)**	25.3 ± 56.0	31.6 ± 69.5	19.8 ± 40.1	33.7 ± 78.0 **	49.0 ± 103 **	20.5 ± 42.3
**Consumers (%)**	59.1	66.6	52.2	54.2	59.3	49.6
**Consumer intake (g) ^#^**	42.9 ± 67.6	47.7 ± 80.8	37.7 ± 48.9	62.6 ± 97.4	82.4 ± 123	41.9 ± 52.5
**Total cheese (g)**	20.7 ± 17.6	20.5 ± 18.8	20.8 ± 16.5	24.2 ± 28.3 **	26.0 ± 29.4 **	22.5 ± 27.1
**Consumers (%)**	98.4	98.5	98.3	96.0 **	96.7	95.4 *
**Consumer intake (g) ^#^**	21.0 ± 17.6	20.8 ± 18.8	21.2 ± 16.4	25.1 ± 28.4	26.9 ± 29.5	23.6 ± 27.2
**Regular cheese (g)**	11.2 ± 15.0	11.3 ± 17.7	11.1 ± 12.2	13.1 ± 21.1 **	14.6 ± 21.6 **	11.9 ± 20.5
**Consumers (%)**	75.7	74.4	76.8	72.3 **	73.2 **	71.6 **
**Consumer intake (g) ^#^**	13.5 ± 14.5	14.6 ± 17.2	12.5 ± 11.8	18.2 ± 22.9	20.0 ± 23.1	16.6 ± 22.6
**Reduced fat cheese (g) (including cottage cheese)**	6.52 ± 10.9	5.85 ± 9.40	7.09 ± 11.9	7.72 ± 17.1 *	7.19 ± 16.4	8.19 ± 17.7
**Consumers (%)**	47.6	45.6	49.2	43.4 *	38.1 *	47.9
**Consumer intake (g) ^#^**	9.4 ± 12.7	8.5 ± 10.6	10.0 ± 14.2	17.8 ± 22.3 **	18.9 ± 22.2 **	17.1 ± 22.5 **
**Cheese from pizza (g)**	2.7 ± 2.6	3.0 ± 3.0	2.4 ± 2.2	3.0 ± 4.2 **	3.9 ± 5.3 **	2.3 ± 2.8
**Consumers (%)**	86.5	87.0	86.1	81.7 **	86.7	77.4 **
**Consumer intake (g) ^#^**	3.1 ± 2.6	3.4 ± 2.9	2.7 ± 2.2	3.7 ± 4.4	4.5 ± 5.5	3.0 ± 2.8
**Total yoghurt (g)**	50.5 ± 62.9	52.9 ± 66.2	48.4 ± 59.9	35.5 ± 59.2 **	36.0 ± 60.3 **	35.0 ± 58.4 **
**Consumers (%)**	73.6	71.9	75.1	60.5 **	58.6 **	62.0 **
**Consumer intake (g) ^#^**	68.7 ± 64.3	73.6 ± 67.6	64.5 ± 61.2	58.7 ± 66.6	61.5 ± 68.1	56.5 ± 65.4
**Regular yoghurt (g)**	16.9 ± 43.5	18.3 ± 49.1	15.7 ± 38.1	12.7 ± 42.7 *	16.5 ± 48.6	9.36 ± 36.5 **
**Consumers (%)**	26.6	25.8	27.3	21.2 **	26.1	16.9 **
**Consumer intake (g) ^#^**	63.6 ± 64.6	70.9 ± 75.2	57.6 ± 53.9	60.1 ± 76.1	63.6 ± 78.2	55.3 ± 73.4
**Reduced fat yoghurt (g)**	33.4 ± 56.5	34.3 ± 56.8	32.6 ± 56.3	22.7 ± 47.6 **	19.2 ± 43.6 **	25.7 ± 50.6 *
**Consumers (%)**	46.5	45.4	47.5	39.1 **	32.3 **	44.9
**Consumer intake (g) ^#^**	71.8 ± 64.2	75.6 ± 63.3	68.7 ± 64.8	58.1 ± 61.1	59.5 ± 59.2	57.2 ± 62.4
**Dairy from milk dishes ^a^ (g)**	3.2 ± 8.1	3.7 ± 9.7	2.7 ± 6.384	2.6 ± 7.8	3.3 ± 9.5	1.9 ± 6.00
**Consumers (%)**	31.2	34.1	28.6	22.9 **	25.3 **	20.8 **
**Consumer intake (g) ^#^**	10.2 ± 11.8	10.9 ± 4.0	9.46 ± 8.87	11.2 ± 13.1	12.9 ± 15.2	9.35 ± 10.2
**Total ice cream (g)**	17.6 ± 15.1	18.8 ± 14.8	16.4 ± 15.3	12.4 ± 13.9 **	14.1 ± 16.1 **	11.0 ± 11.5 **
**Consumers (%)**	96.1	96.9	95.4	88.1 **	87.4 **	88.8 **
**Consumer intake (g) ^#^**	18.2 ± 14.9	19.4 ± 14.5	17.1 ± 15.2	14.1 ± 13.9	15.9 ± 16.2	12.5 ± 11.5
**Regular ice cream (g)**	15.3 ± 14.6	16.3 ± 14.4	14.5 ± 14.9	10.9 ± 13.6 **	12.8 ± 15.8 **	9.34 ± 11.1 **
**Consumers (%)**	94.5	95.7	93.5	85.6 **	85.5 **	85.7 **
**Consumer intake (g) ^#^**	16.2 ± 14.6	17.0 ± 14.3	15.5 ± 14.8	12.8 ± 13.9	14.9 ± 16.1	10.9 ± 11.2
**Reduced fat ice cream (g)**	2.3 ± 6.9	2.6 ± 7.2	2.0 ± 6.6	1.5 ± 4.1 **	1.3 ± 4.3 **	1.7 ± 3.8
**Consumers (%)**	27.6	27.3	27.8	28.6	23.3	33.2
**Consumer intake (g) ^#^**	8.3 ± 11.1	9.4 ± 11.3	7.3 ± 10.9	5.3 ± 6.1	5.6 ± 7.5	5.1 ± 5.1
**Butter (g)**	4.8 ± 10.5	5.5 ± 11.6	4.2 ± 9.5	7.2 ± 12.1 **	8.8 ± 14.6 **	5.7 ± 9.3 **
**Consumers (%)**	21.2	22.6	20.0	36.7 **	38.1 **	35.6 **
**Consumer intake (g) ^#^**	22.5 ± 11.1	24.3 ± 11.5	20.8 ± 10.4	19.5 ± 12.6	23.2 ± 15.0	16.1 ± 8.7
**Cream (g)**	0.9 ± 2.3	0.9 ± 2.1	0.9 ± 2.5	0.8 ± 4.0	0.8 ± 5.4	0.7 ± 2.1
**Consumers (%)**	34.9	35.6	34.3	24.5 **	23.6 **	25.4 **
**Consumer intake (g) ^#^**	2.7 ± 3.3	2.6 ± 2.9	2.7 ± 3.7	3.2 ± 7.6	3.6 ± 10.7	2.8 ± 3.3
**Custard (g)**	4.9 ± 12.1	6.5 ± 15.2	3.4 ± 8.3	3.8 ± 11.4 *	4.8 ± 13.5	2.9 ± 9.07
**Consumers (%)**	37.6	42.4	33.4	27.7 **	31.3 **	24.5 **
**Consumer intake (g) ^#^**	13.0 ± 16.9	15.2 ± 20.3	10.6 ± 11.6	13.6 ± 18.4	15.2 ± 20.8	11.8 ± 15.2

* *P* < 0.05 significant difference from 14 year, ** *P* < 0.01 significant difference from 14 year; ^#^ Significance not calculated for consumer intake in grams as subjects differed between age categories; ^a^ Milk dishes refers to milk pudding and mornay dishes.

### 3.4. Intake of Dairy Products by Serves

**Figure 1:**
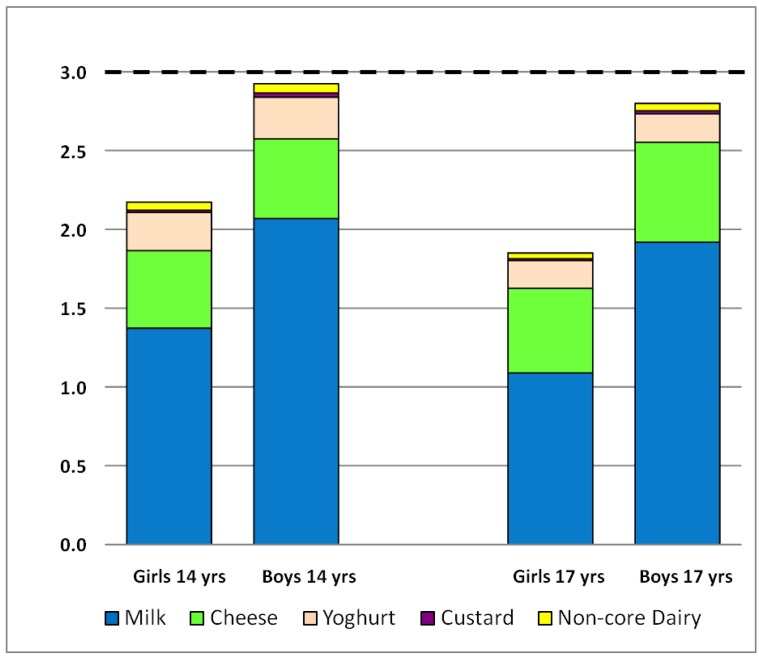
Mean serves of dairy per day for girls and boys at 14 and 17 years, split by type. Core dairy products are milk (including flavoured milk and smoothies), yoghurt, cheese and custard. Non-core dairy products are butter, ice cream and cream adjusted to calcium serves of 300 mg. The dashed line represents the recommended minimum three serves a day guideline for adolescents [33].

Figure 1 shows the mean intake of core and non-core dairy product serves per day for boys and girls from 14 to 17 years, compared with the recommended three serves per day for these age groups [33]. Girls at 17 years reported the lowest mean intake with 1.85 serves, followed by girls at 14 years with 2.17 serves. Boys at 14 years reported the highest intake with 2.92 serves, with boys at 17 years reporting 2.80 serves. Milk was the largest contributor to daily serves for both genders and age groups, followed by cheese and yoghurt. Non-core dairy products accounted for 1% or less of daily serves on a matched calcium basis. At 14 years, 21.5% of girls and 40.1% of boys met the 3 serves/day guideline. At 17 years, this decreased to 13.9% of girls and 36.6% of boys.

### 3.5. Gender, BMI and SES as Predictors of Change in Dairy Intake

**Figure 2:**
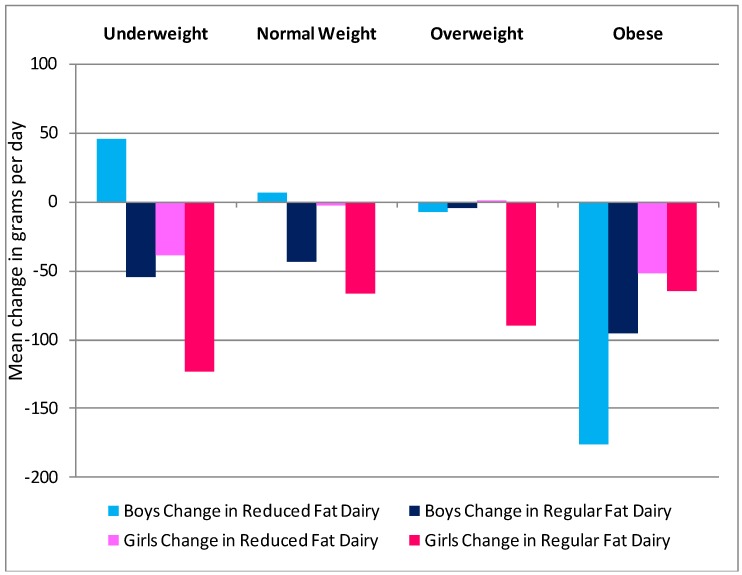
Mean change in grams of total, reduced fat and regular fat dairy in grams per day in Raine Study participants from 14 to 17 year follow-ups, split by body mass index (BMI) categories at 14 years [28,29].

**Figure 3:**
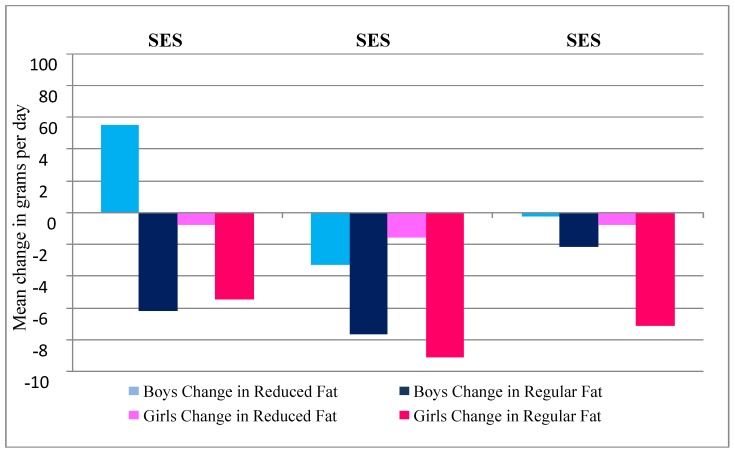
Mean change in grams of total, reduced fat and regular fat dairy in grams per day in Raine Study participants from 14 to 17 year follow-ups, split by socioeconomic status (SES) at 14 years as determined by Socio-Economic Indexes for Areas [30].

Although both genders decreased mean daily dairy intake from 14 to 17 years, girls compared to boys reported greater decreases in total dairy (87 g *vs.* 52 g, *P* = 0.13), reduced fat dairy intake (13 g *vs*. 7 g, *P* = 0.78) and regular fat dairy intake (75 g *vs.* 45 g, *P* = 0.13). Dairy intake decreased from 14 to 17 years across all weight status categories. Adolescents who were overweight significantly decreased their intake by an average of 190 g between 14 and 17 years of age (*P* = 0.048). Boys who were underweight or normal weight were the only ones to show an increase in dairy (reduced fat), and this was significantly increased compared with the obese group (*P* = 0.044) (Figure 2). No significant differences over BMI categories were observed for girls. There were also no statistically significant differences in change in intake over SES category, although boys from the low SES areas were the only group to report a mean increase in dairy intake (reduced fat) (Figure 3). 

## 4. Discussion

Results of our study indicate that on average, adolescents decrease their dairy intake from 14 to 17 years. As a consequence, our findings suggest that nutrient intakes are less than optimal during the important adolescent growth period. Based on an average serve of dairy contributing 300 mg of calcium, from our study results we estimate that dairy products contribute 65% of calcium intake for girls and 68% for boys at 14 years, decreasing slightly to 61% for girls and 65% for boys at 17 years. The majority of our adolescents did not meet the RDIs for calcium and magnesium at 14 years, and this situation worsened as they became older. Suboptimal calcium intake has been previously identified in Australian adolescents [21,37], with girls showing lower intakes than boys [21]. Our results are also similar to a longitudinal study conducted in the United States, which showed that teenagers reduced their calcium intake from the ages of 16 to 21, particularly females [38]. 

These findings are of concern for both genders, as adequate calcium intake during adolescence is important for bone growth, development and for reducing the risk of bone fractures during adolescence, as well as laying the foundation for bone health later in life [10,39,40,41]. Females in particular are at risk of osteoporosis and hip fracture, due to the loss of the bone protective factor oestrogen during menopause and older age [1,39]. In addition to calcium being of concern, magnesium is also an important nutrient as a cofactor for over 300 enzymes involved in metabolism of food and synthesis of metabolic products [42]. Associations have been found between magnesium deficiency and insulin resistance in obese children [43], and there are links with osteoporosis [11,44] and diabetes [42,45]. The link with insulin resistance and diabetes is thought to be related to the use of magnesium in carbohydrate metabolism [42]. Magnesium is thought to contribute to bone health through affects on parathyroid hormone secretion, with low intakes of magnesium leading to lower parathyroid hormone and a decrease in serum 1,25 dihydroxy vitamin D3, resulting in altered hydroxyapitite crystal formation and impaired bone health [42]. In our cohort, mean intakes for dairy products fell short of the recommended three serves per day [33] for girls at both age groups, with boys closer to but still less than three. Our figures of between 14% and 22% of girls and 37% and 40% of boys meeting recommendations supports previously reported figures of 17% for girls and 38% of boys in the Australian adolescent population overall [46]. Depending upon an individual’s activity level and energy requirements, some adolescents, particularly boys, may benefit from up to five serves of dairy per day [33]. 

We observed in our study that lower milk consumption was responsible for the largest decrease as the adolescents grew older. Total dairy intake, and that of most dairy products, decreased from 14 to 17 years for both boys and girls. The exceptions were cheese and butter, which increased significantly over this period. 

To compare our results with other Australian research, the 1995 National Nutrition Survey reported cheese intake along with butter/dairy fat intake was higher in 16–18 years old compared with 12–15 years old [47], although the total dairy category of milk products and dishes was also shown to be higher in the older age group. The National Nutrition Survey is an older study and therefore it is possible there has been some change in habits and eating patterns since the time of data collection. The more recent Child and Adolescent Physical Activity and Nutrition Survey of Western Australia surveyed primary and secondary school students in 2003 and 2008, and found the proportion of students consuming milk products and dishes decreased from primary to secondary school, and from 2003 to 2008, significantly so for girls [21]. These results are similar to our findings, although this survey used a different group of adolescents at each time point (2003 and 2008), whereas our study followed the same adolescents. The 2007 Australian National Children’s Nutrition and Physical Activity Survey also showed gender differences. Total milk intake was lower in girls in the 14–16 year age group compared to the 9–13 age group, but the opposite was reported for boys [24]. This survey also showed mean intake of flavoured milk was higher in older boys, similar to the trend we observed in our adolescents. This trend may be due in part to marketing campaigns targeting the male audience, and the increasing overall popularity of flavoured milk (usually fat reduced), particularly iced coffee [48]. We found in the Raine Study that boys from the low SES areas were the only group to report a mean increase in dairy intake. We postulate that boys from lower SES backgrounds may be more likely to drop out of school and enter apprenticeships as blue collar workers [49], and tradesmen may have more of a culture of consumption of flavored milks like iced coffee. An Australian product development company refers to market research describing the primary consumers of iced coffee as stalwart tradesman who would consume several cartons daily, often referring to it as “daytime beer” [50].

In terms of predicting change in dairy intake from 14 to 17 years, our results showed that weight category was a significant predictor. Adolescents who were overweight at 14 years were more likely to have decreased their dairy intake at 17 years, by an average of 190 g/day. Decreasing dairy intake may be a result of trying to lose weight, particularly in girls [20,51]. The category of underweight boys was the only weight category to increase consumption from 14 years, and this may reflect trying to build body mass or a lack of concern over weight gain related to dairy intake, whether this stigma is justified or not. Recent evidence suggests that it is not justified, with a review of prospective cohort studies suggesting that the consumption of dairy foods may actually reduce the risk of overweight, rather than increase [52]. The review found that three out of ten studies in children and adolescents showed protection against weight gain, with one suggesting an increased risk and six studies showing no association. In adults, a meta-analysis of randomized controlled trials found an overall negative association between weight gain and dairy intake which bordered significance, with the combination of increased dairy with energy restriction significantly associated with a decrease in weight [53]. 

Our study is unique as it follows the same large population based sample of adolescents from 14 years to 17 years to allow matched pairing of subjects for analysis of changes in dairy consumption over time. This study also provides valuable information for further studies of calcium intake and bone status in the same cohort. The FFQ method for analysing dietary intake has the advantage of being cost effective given the large subject group size and with a relatively low subject burden. A potential limitation of the CSIRO FFQ was that quantity information was generated from frequency data in relation to standard serve sizes. This required the respondent to consider their usual serve size in comparison to the standard given and then adjust the reported frequency accordingly, which may increase the risk of estimation error. Although the accuracy of FFQs can be of concern [54], the FFQ used has been validated in our Raine Study group with a three day food record [55]. The power of analyses investigating intakes by BMI and SES categories were limited by small numbers in some groups. Although Raine Study adolescents participating in dietary studies tend to have older mothers, higher family income or a lower BMI than the non-respondents [56], families who were involved in the study were more likely to be of middle to lower socioeconomic status initially [57], which may improve the generalisation of our results.

## 5. Conclusions

Our findings identify a discernible gap between reported consumption and recommended nutrient and dairy product intake over the adolescent period, particularly for girls. Possible barriers for adolescents consuming dairy products may include lactose intolerance, changes in eating patterns with a changing lifestyle (for example skipping breakfast), and being conscious of their weight and body image. Increasing intake of dairy products will help improve intakes of a range of important nutrients, particularly calcium and magnesium that were especially lacking compared to recommended intakes in our subject group. A recent review identified taste exposure and prompting practice as important strategies utilised in effective interventions to increase calcium or dairy intake, along with concentrated delivery across a variety of settings [58]. 

Our results suggest that both girls and boys are reducing their intake of dairy foods at a time when calcium and other nutrient requirements are increased. Furthermore, the majority of adolescents are not meeting their calcium requirements at early adolescence, and this continues into later adolescence.Adolescence is considered an important time for physiological growth and prevention of future chronic disease such as osteoporosis [11,44] and diabetes [45]. Based on our findings, public health messages for this age group may benefit from promotion of a calcium rich food at every meal, such as dairy products. Non-dairy foods that are good sources of calcium include sardines and salmon (with bones), broccoli, nuts and seeds, and products fortified with calcium such as breakfast cereals, and soy beverages. It may be more beneficial to obtain calcium from food sources than supplements, due to suggestions that high dosage supplements may have health consequences such as kidney stones and artery calcification which could lead to heart disease [59]. Future research investigating reasons behind the decline we have observed in our population would help further inform public health campaigns addressed at improving healthy eating during adolescence.
